# Supplementary material for: Modelling the dynamics of senescence spread
Source: Aging Cell. 2023 Jun 8;22(8):e13892. doi: 10.1111/acel.13892 (PMC10410058; doi:10.1111/acel.13892)
Supplement: Supplementary file 1 — Figure S1 [file ACEL-22-e13892-s001.docx]

# Supplementary Information

**a**
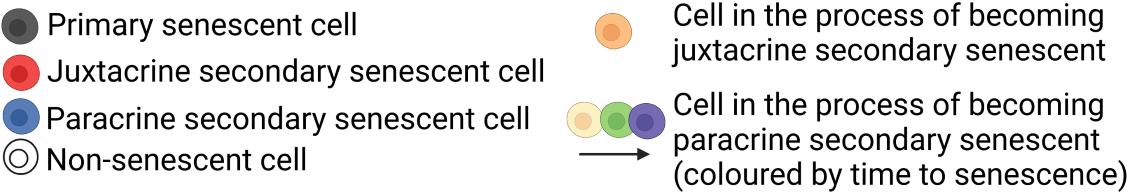


No time delay


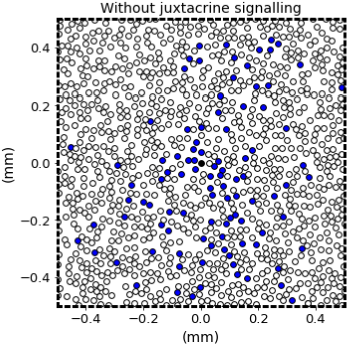


**b**


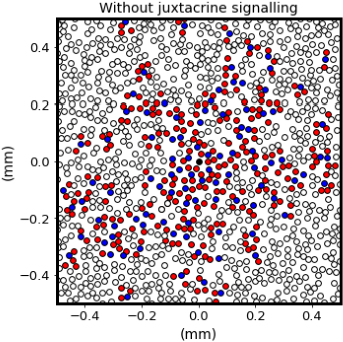

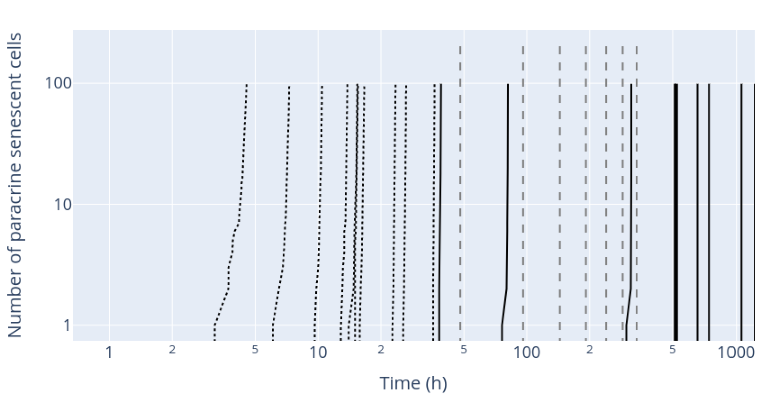

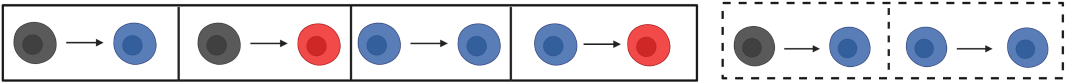


## Time delay on paracrine transmission and juxtacrine transmission


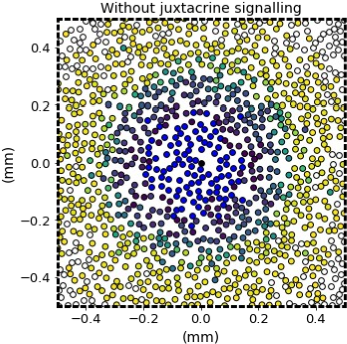


**c**


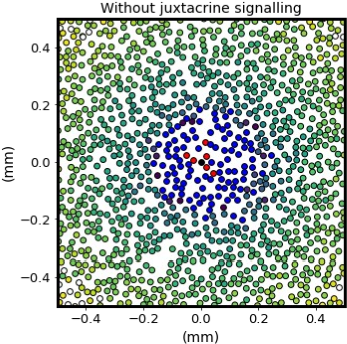

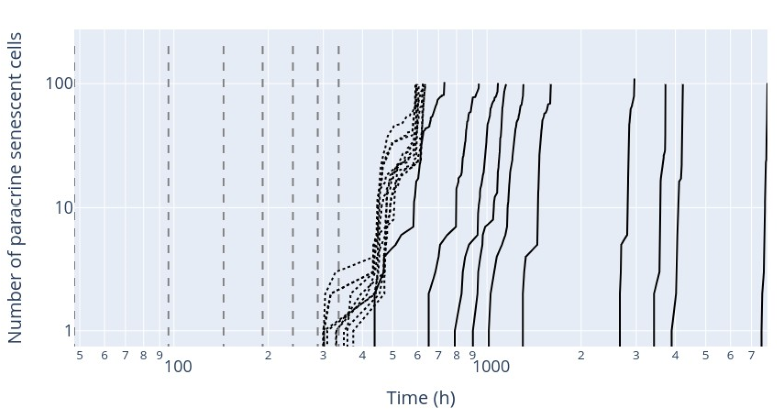

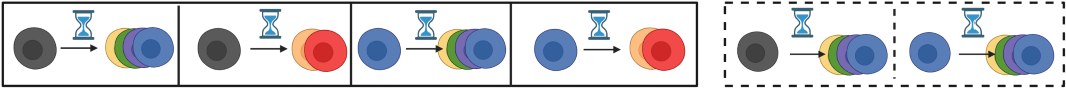


Time delay on paracrine transmission


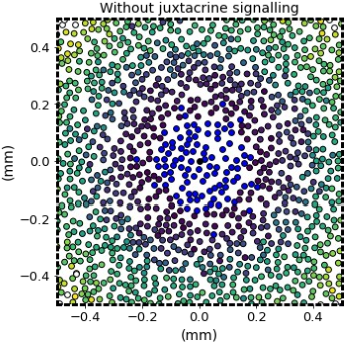

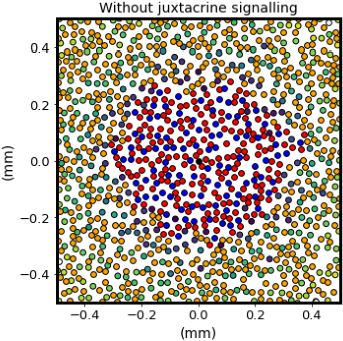

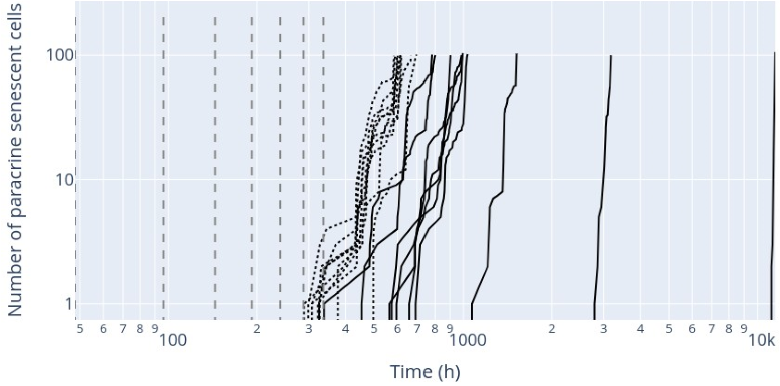

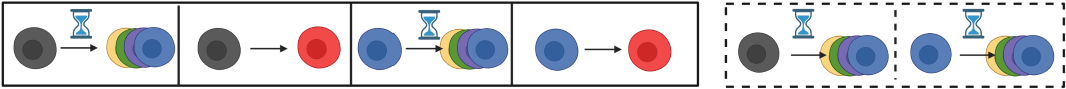


Figure S1:

Figure S1: a) The spread of senescence from a single primary senescent cell with *R*_tot_ = 10^5^, *σ* = 0*.*35, *k_e_* = *k_off_* = 0*.*2, *N*_D_ = 9, and no delay in the induction of senescence. The left-hand side plots show the spatial distribution of senescent cells and the right-hand side shows the creation of paracrine secondary senescent cells over time, both with juxtacrine senescence (solid) and without (dotted). Vertical grey dashed lines mark days 1-7. b) The same simulation as in (a), but with a 6-day time delay before a cell can induce senescence in its neighbours.

c) The same simulation as in (b), but with immediate induction of juxtacrine senescence and delayed induction of paracrine senescence.


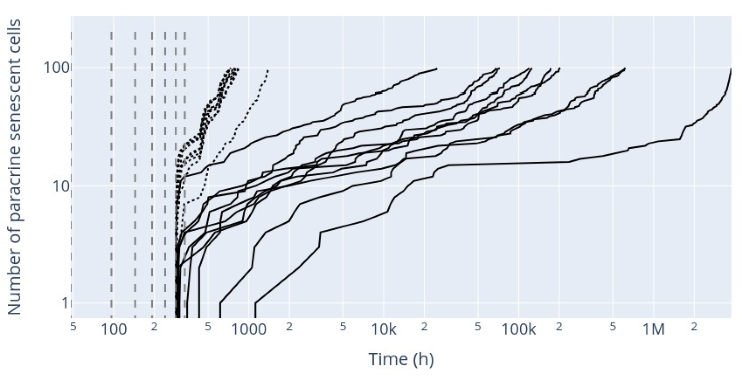

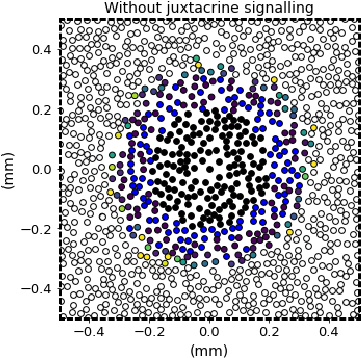

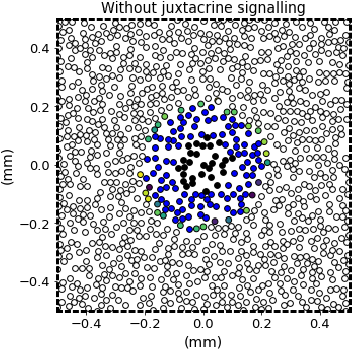


**a**


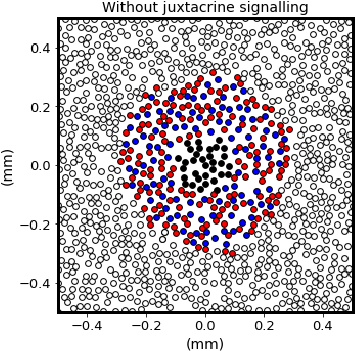


Time delay on paracrine transmission, half strength SASP

**b**

Time delay on paracrine transmission, half strength SASP, double lesion radius


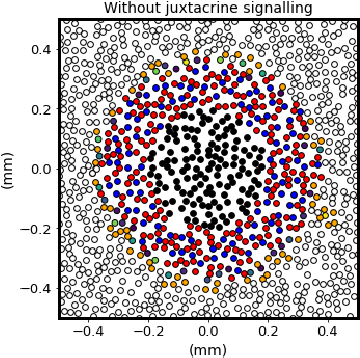

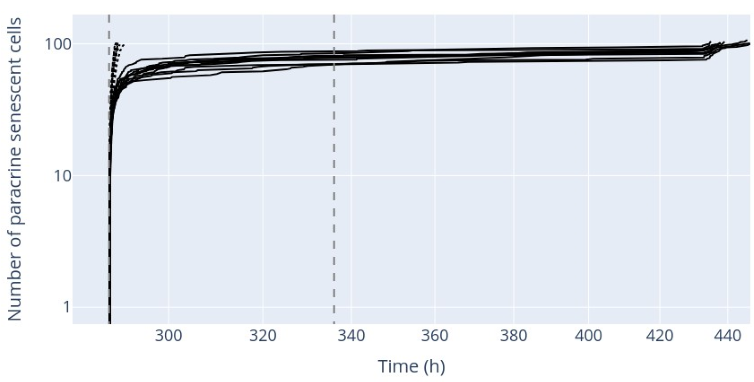

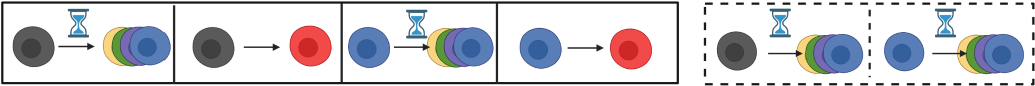

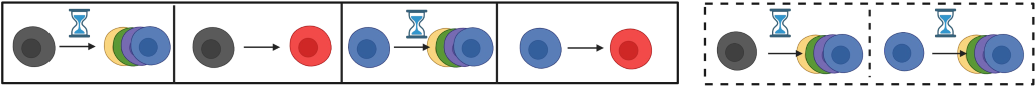


Figure S2: a) The spread of senescence from a lesion of primary senescent cells with *R*_tot_ = 10^5^, *σ* = 0*.*35, *k_e_* = *k_off_* = 0*.*2, *N*_D_ = 62, and a 6-day delay in the induction of senescence. In this simulation, paracrine secondary senescent cells produce half of the SASP of primary senescent cells. The vertical purple line highlights 100,000 hours (≈ 11 years), simulations which cross this line show control of senescence over a human lifespan. d) The same simulation as in (c), but from a larger primary lesion.

**a**


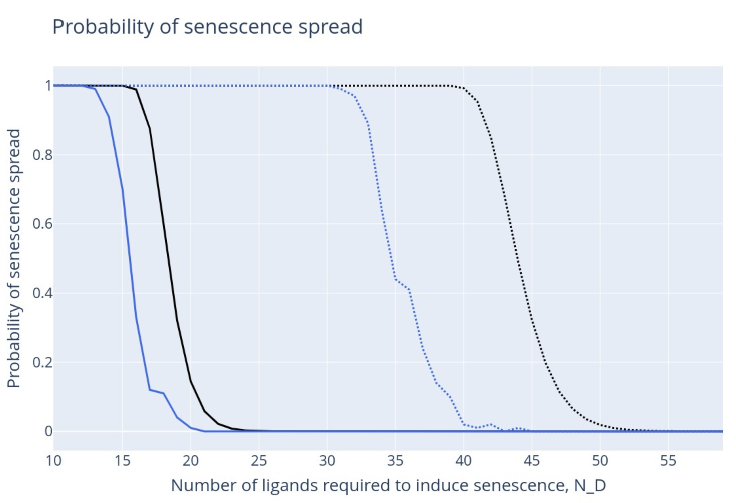


**b**


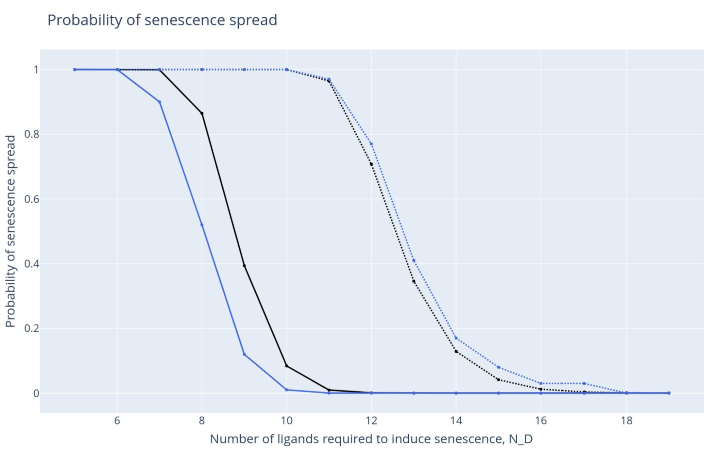

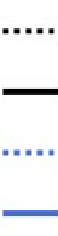

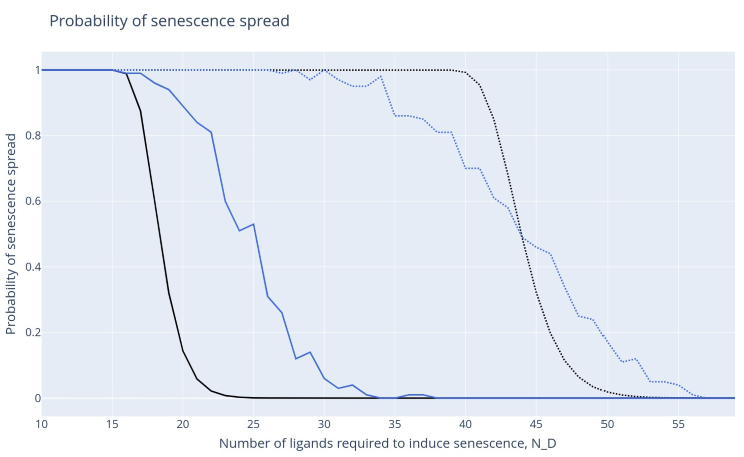


**c**

Numeric no juxtacrine

Numeric with juxtacrine

Stochastic no juxtacrine Stochastic with juxtacrine

**d**


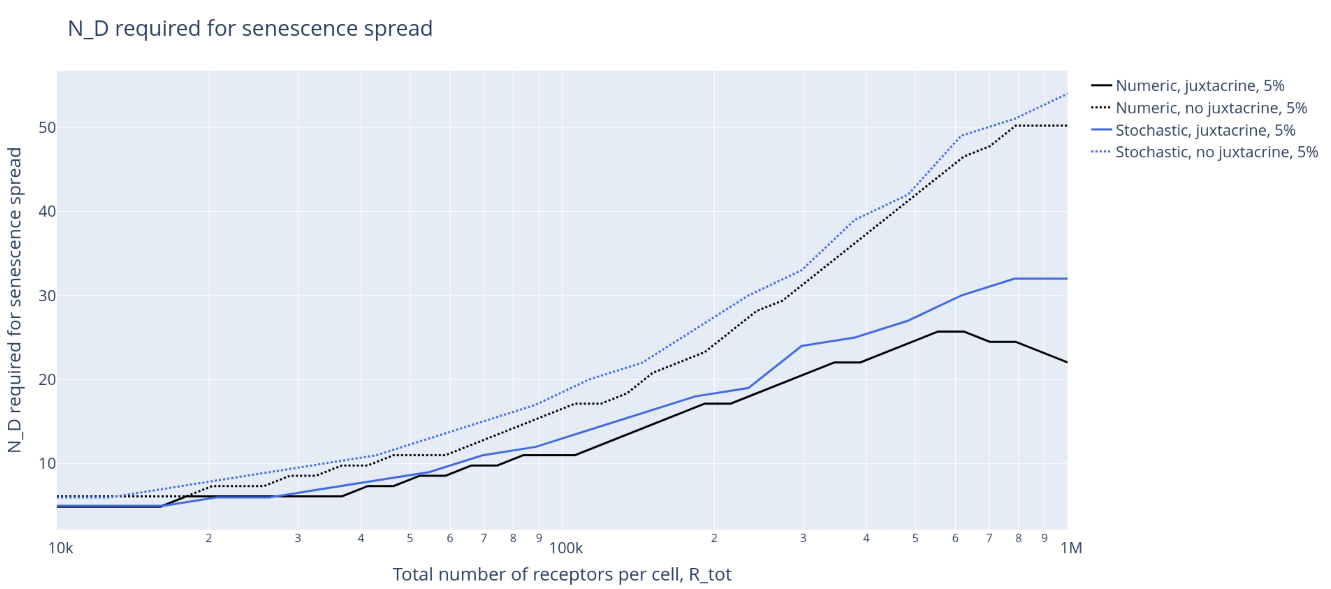


Figure S3:

Figure S3: Comparison of the stochastic simulation to the minimal model. In (a) - (d) the stochastic simulation was run 100 times for a single primary senescent cell and the probability of senescence spread in 2 days against the number of ligands required to induce senescence. a) *R*_tot_ = 10^5^, density= 0.35, *k_e_* = *k_off_* = 0*.*2, *k_on_* = 10^8^, cells are constrained to a lattice. b) A repeat of the simulation in (a) with *R*_tot_ = 10^6^. c) A repeat of (b), but cells are not constrained to a lattice. d) A comparison of the *N_D_* values required to see 5% senescence spread from a single cell in the minimal model and the stochastic simulations.

**a**


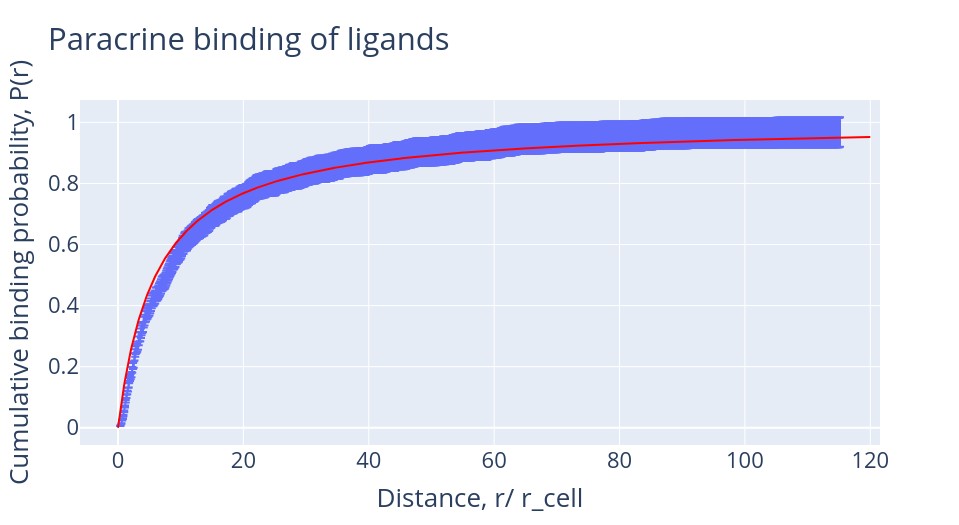

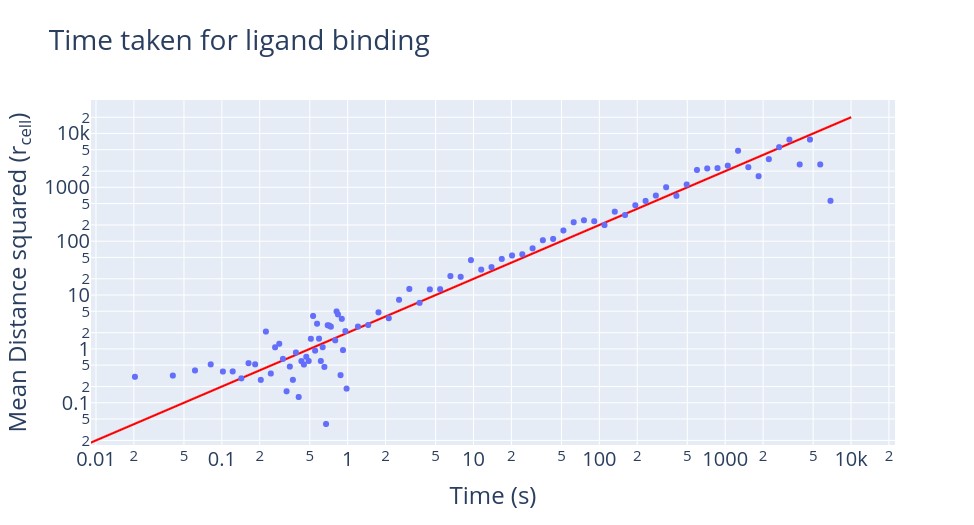

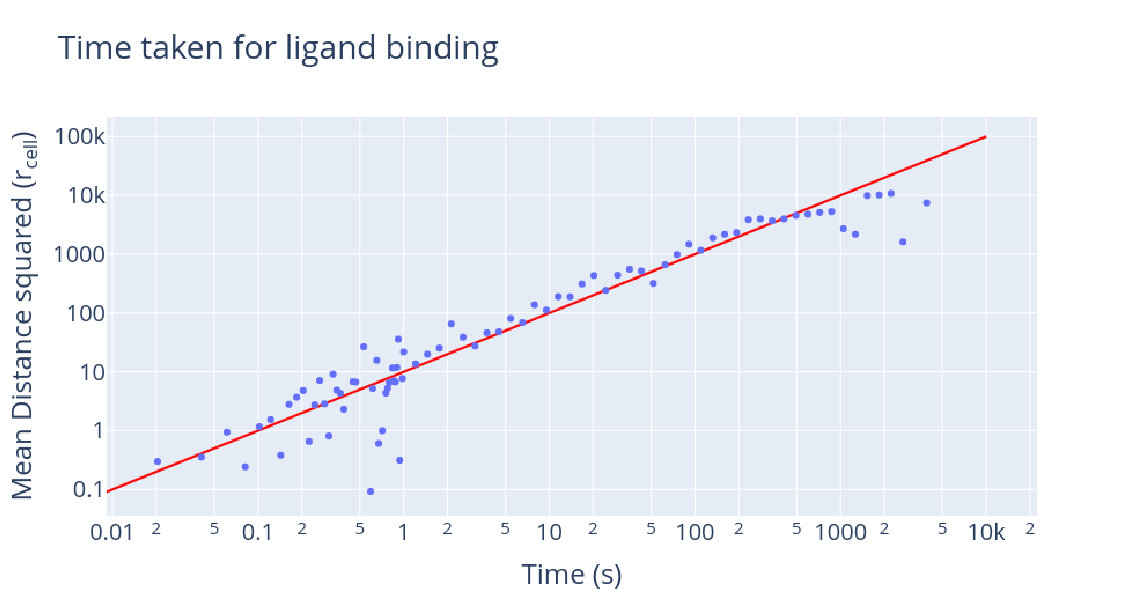


**b**

**c**

Figure S4:

Figure S4: Results from the recreation of the ligand binding code described by Batsilas et al, in (Batsilas et al., 2003). 1000 simulations were run in which a ligand was tracked as it was emitted from a cell until the first time it bound to a cell. a) A comparison of the cumulative binding observed in the simulation with a 5% error bar (blue) and equation 1 (red). Both (b) and (c) show the results of these simulations, plotting the mean distance at which a ligand bound against the time it took for this binding to occur. The blue points show the simulation result, and the red line the expected result if this was a diffusion-limited process. a & b) *D*_L_ = 10^−6^ cm^2^/s, cell density = 0.2, media height = 0.002 m, *R*_tot_ = 10^6^, and *k_on_* = 10^8^. b) *D*_L_ = 5×10^−6^ *cm*^2^*/s*, cell density = 0.35, media height = 0.002 m, *R*_tot_ = 10^5^, and *k_on_* = 10^8^.

**b**

**a**


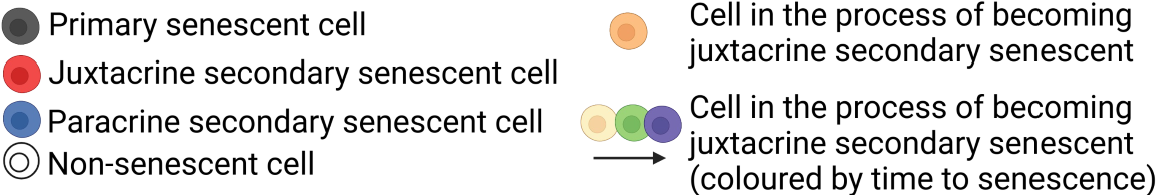

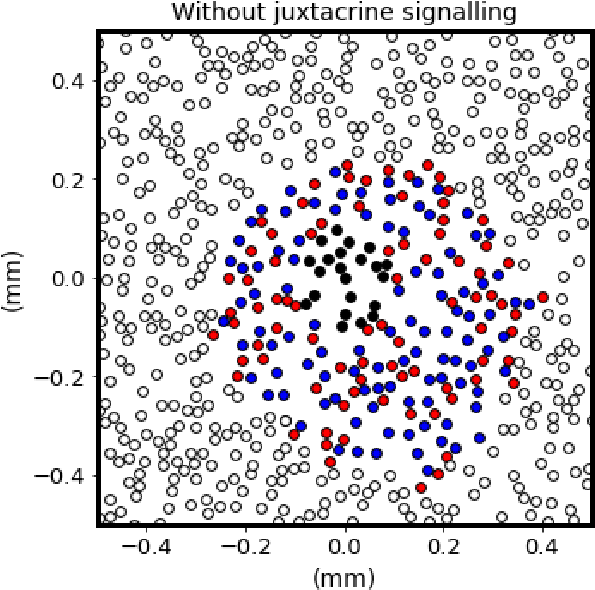

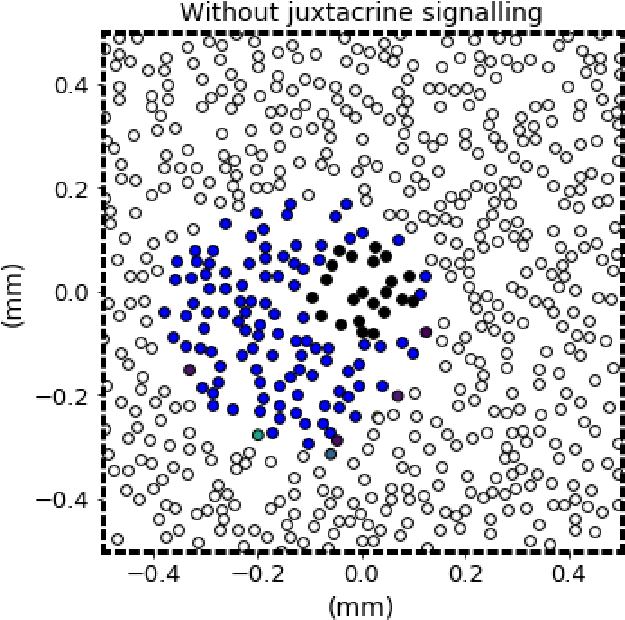


**^c^** With delayed juxtacrine signaling **^d^** With instantaneous juxtacrine signaling


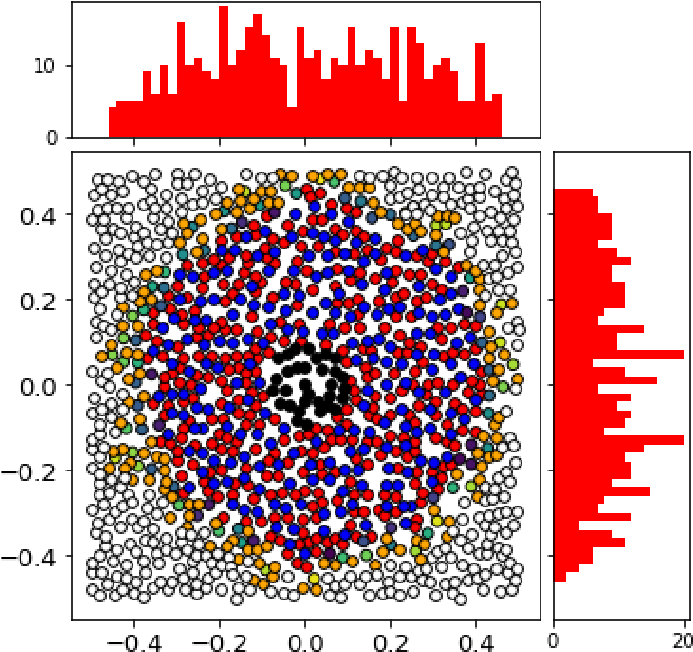

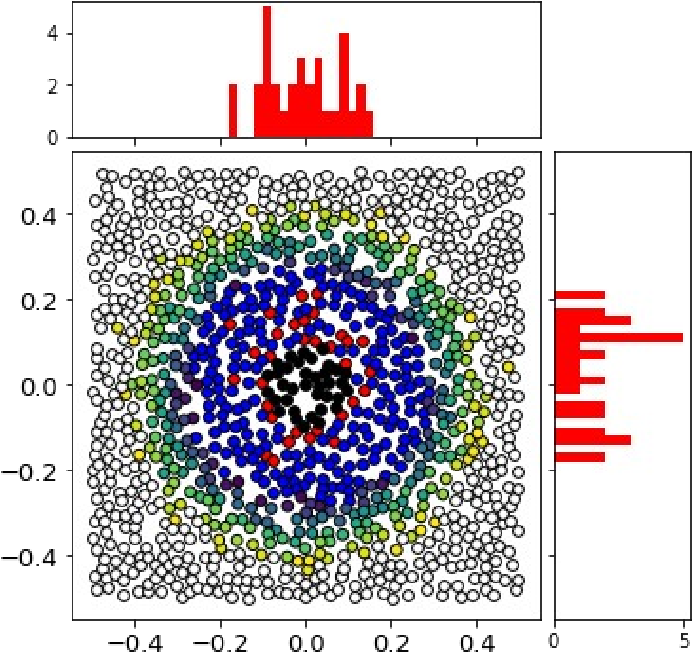


Figure S5: (a) and (b) show a repeat of the simulation in figure 3e, with the reduced density of 0.2. (c) and (d) show senescence spread from a lesion both with and without a delay in the juxtacrine signalling, with histograms showing the density of juxtacrine secondary senescent cells.


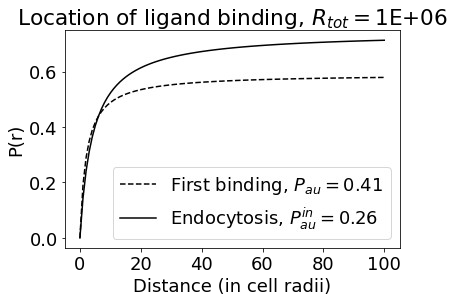

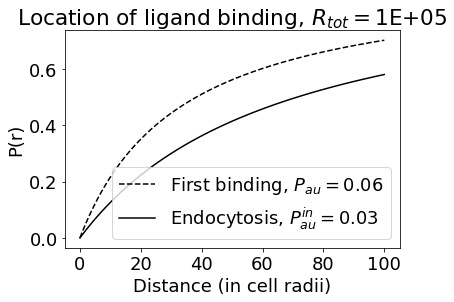


Figure S6: Comparison of the binding and internalisation distances.


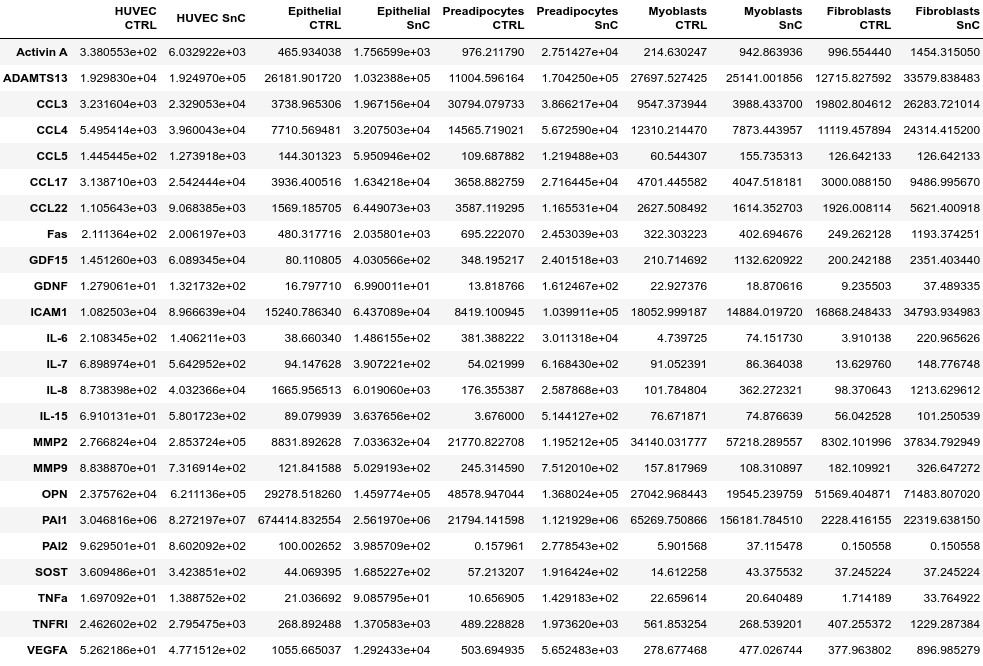


Figure S7: Number of SASP proteins produced per cell per hour, from data in

(Schafer et al., 2020)

**a b**


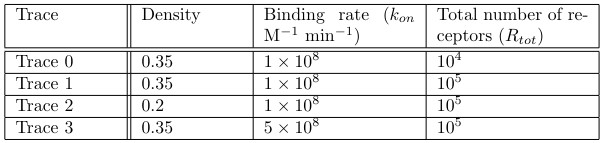

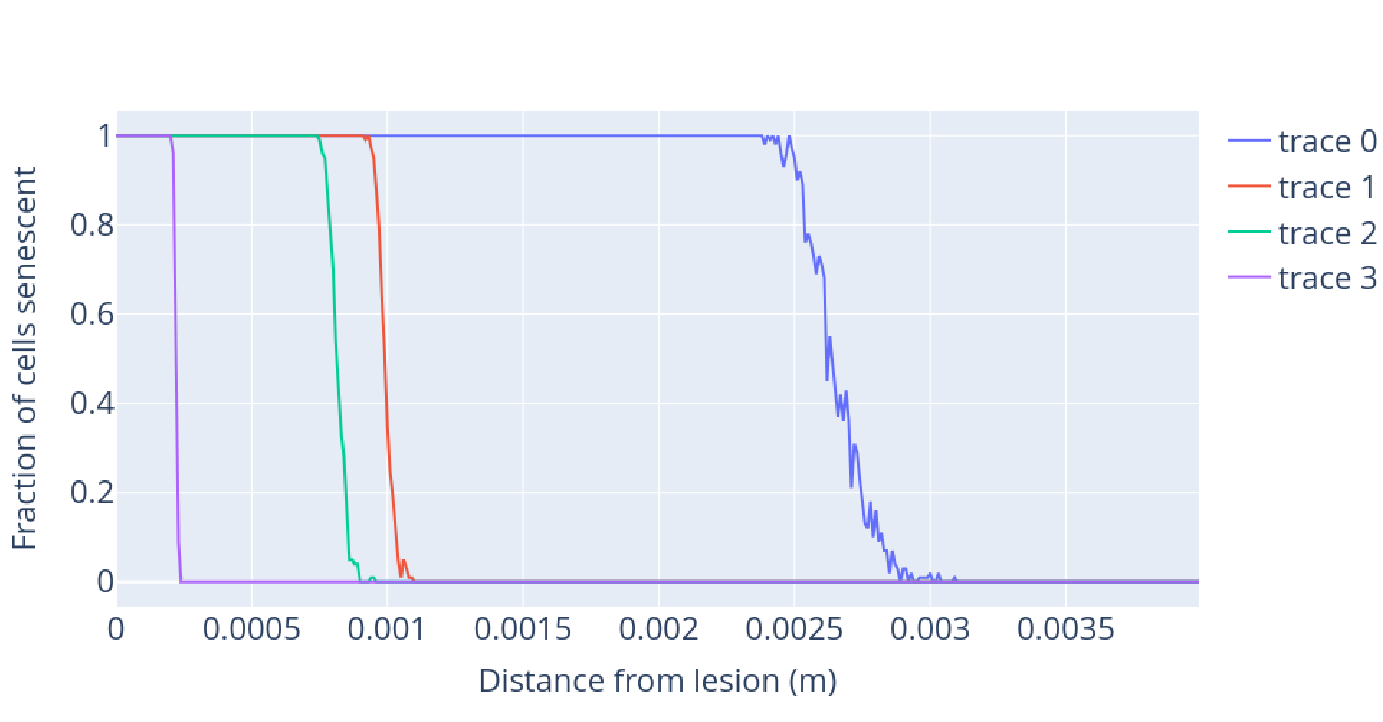


Figure S8: a) Posterior for the 4 inferred parameters, with red lines showing the true parameter values. True parameters were *r*_cell_ = 10 × 10^−6^, density = 0.35, length of experiment = 2 days, the radius of seeded region = 1000 × *r*_cell_, *N*_E_ = 2887 per hour, and *N*_D_ = 140 per hour.


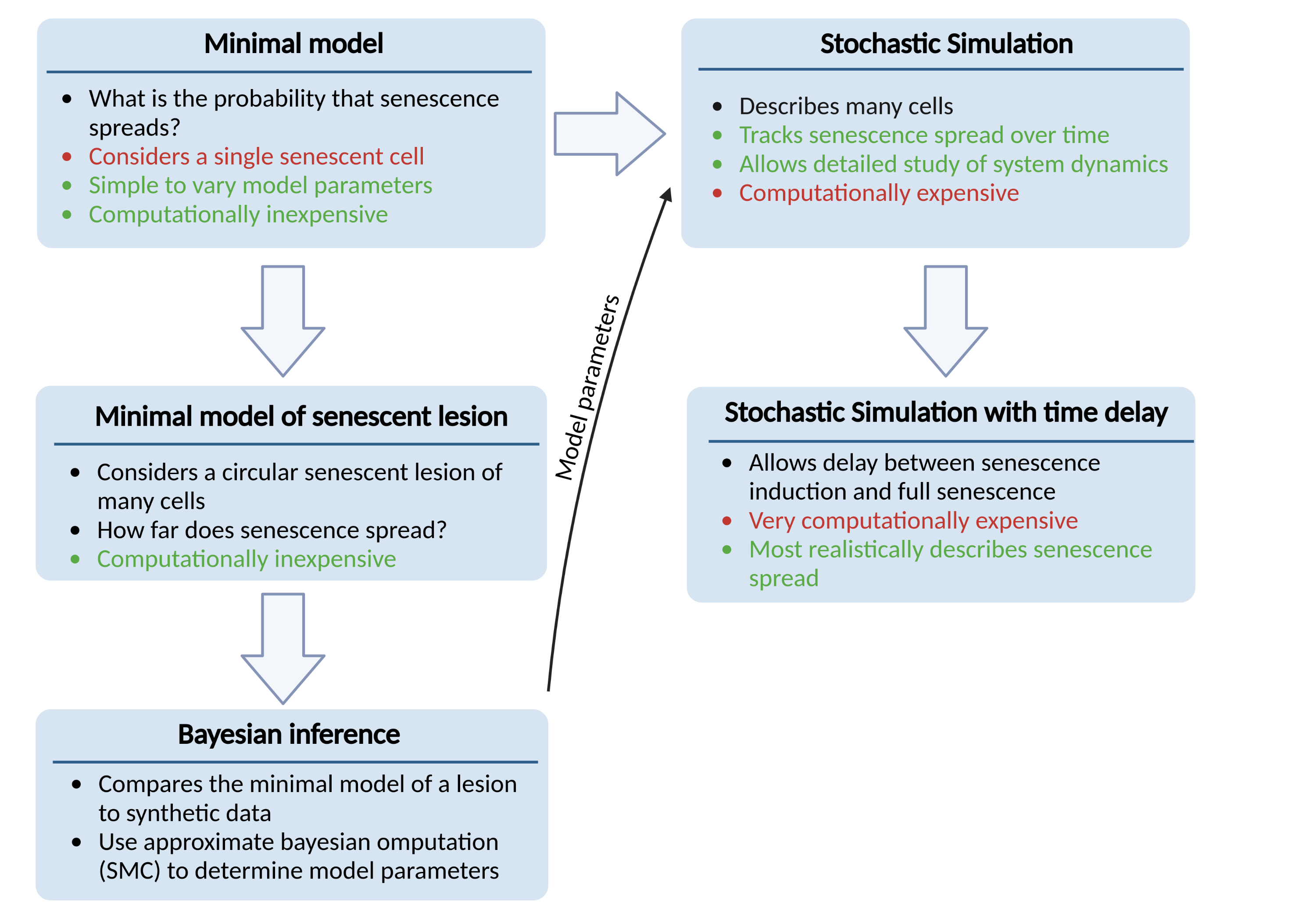


Figure S9: Flowchart showing the relationship between the models of senescence spread discussed in this paper.


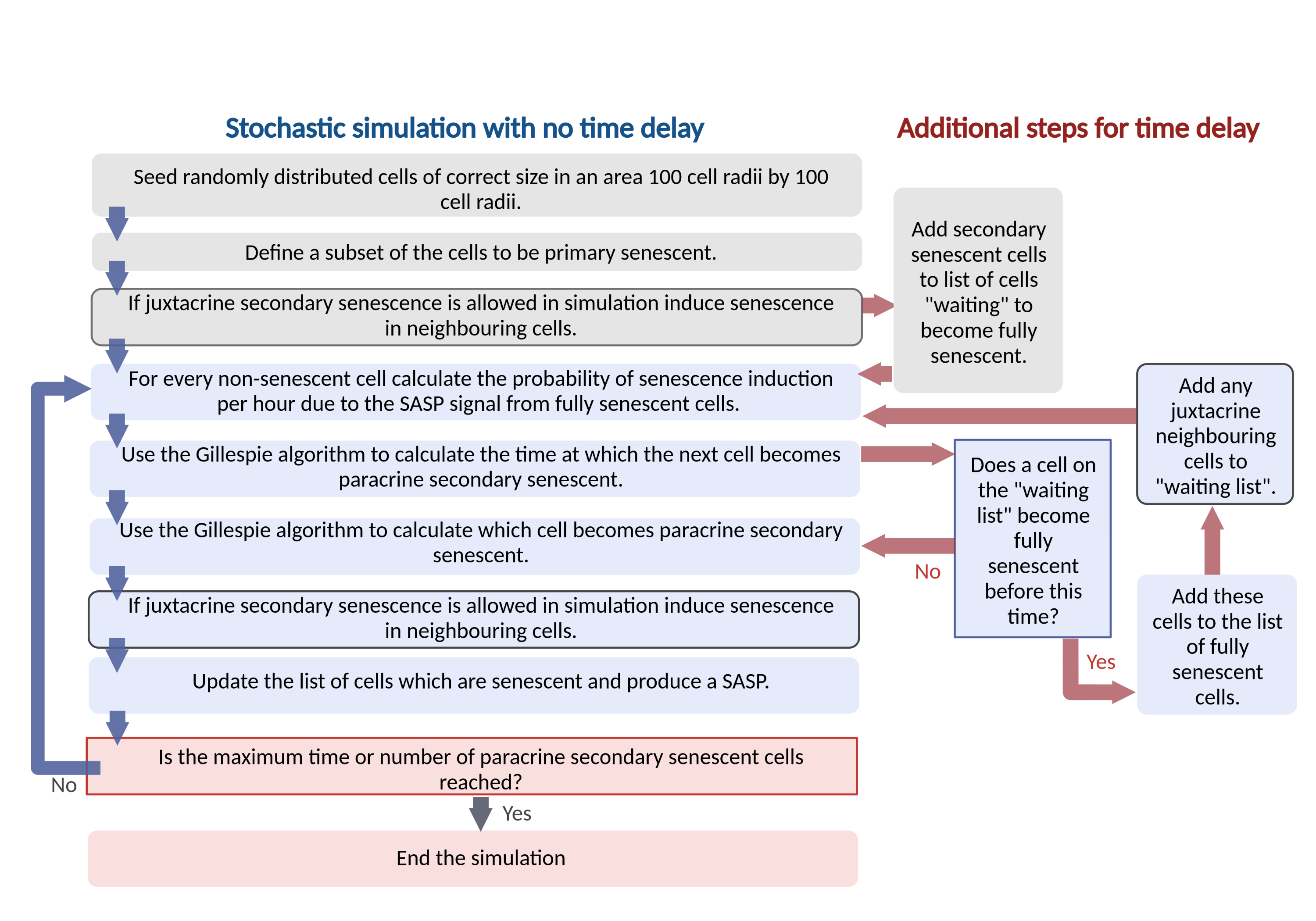


Figure S10: Flow chart (pseudo-code) showing the implementation of the stochastic simulation.
